# Biomarker Studies in Stress Biology: From the Gene to Population, from the Organism to the Application

**DOI:** 10.3390/biology10121340

**Published:** 2021-12-16

**Authors:** Marco F. L. Lemos

**Affiliations:** MARE-Marine and Environmental Sciences Centre, ESTM, Polytechnic of Leiria, 2520-641 Peniche, Portugal; marco.lemos@ipleiria.pt

**Keywords:** cost of tolerance, ecotoxicology, energetics, integrated biomarker response, mechanisms of action, neurotoxicity, oxidative stress

## Abstract

**Simple Summary:**

Ecosystems have been increasingly subject to stressful conditions and thus the need to develop tools capable of assessing their impacts on populations and communities. These effects assessed at higher levels of biological organization tend to reflect the sum of effects on individuals, arising from the effects at the cellular and molecular levels. These lower biological organization levels are more responsive at an early stage, allowing them to be used as early warning endpoints to address environmental stress—biomarkers. In this work, the need to link low to high levels of biological organization is addressed and the challenges and usefulness of biomarkers in a myriad of fields discussed.

**Abstract:**

Endpoints assessed at the population or community level are most often the result of the sum of effects on individuals, arising from the effects at the cellular and molecular levels. Within this framework, these lower biological level endpoints are more responsive at an early stage of exposure, making them potential toolboxes to be used as early-warning markers to address stress. Given this, by linking responses and understanding organisms’ metabolism and physiology, the possibilities for the use of biomarkers in stress biology are vast. Here, biomarker comprehensive examples are given to enlighten the need to link levels of biological organization, and their usefulness for a myriad of fields and applications is presented and discussed.

## 1. Introduction to Stress Biology and Biomarkers

Since the early days of mankind and sky rising after the industrial revolution, freshwater, marine, and terrestrial ecosystems have been subjected to a myriad of contaminants and consequent impacts, prompting the need for the establishment of pollution control regulations and monitoring programs. First focused on chemical and microbiological analysis, it was clear that these alone were not sufficient to monitor and protect aquatic ecosystems, and thus the need to develop tools capable of assessing not only the presence of pollutants in the water, but also, and most of all, their latent effects on organisms, their populations, and communities was paramount [1]. To contribute to the environmental health assessments and eventual impact of pollutants, assemblages and diversity of fish, invertebrates, algae, and macrophytes have been used as bioindicators of pollution and water quality for several years to date [2,3,4,5] and are important elements of government regulations and ecological risk assessment (ERA) (e.g., [6,7]).

By using these high levels of biological organization, this type of assessment is therefore highly robust and relevant, and easy to apply and interpret. Using them, it is possible to evaluate the direct effects of anthropogenic or natural disturbances on the structure and/or function of an actual community of aquatic organisms, which cannot be observed when using lower levels of biological organization [8,9]. However, by addressing populations and communities, the specific causes of the observed effects may not be assessed. These, being longer-term effects, may include a temporal gap between the cause and effects that may be difficult to interpret. Also, the predictive capacity of those measurements is restricted because repeatability is difficult, and they may not clearly distinguish between a polluted site and a naturally impoverished one. Despite the relevance of such a level of organization assessment, the major drawback with this approach is that ecologically important effects such as impairment in growth, behaviour, reproduction, death, or even taxa losses, will have already occurred prior to being detected at the population and community levels [10].

It is in this framework that modern ecotoxicology thrives, as the scientific discipline combining methods of ecology and toxicology to study the effects of environmental stressors, i.e., environmental conditions deviating from species optima, while the relevance of responses is paramount to strengthen this field.

## 2. Linking Levels of Biological Organization

Effects assessed at higher levels of biological organization (populations and communities) are most often the result of the sum of effects on individuals, arising from the effects at the cellular and molecular levels [9]. Given this rationale, these lower biological organization levels are more responsive at an early stage, allowing them to be used as early warning endpoints to address environmental stress.

This way, the information concerning impacts at the molecular level of biological organisation (e.g., transcripts or proteins) may allow for an early-in-time assessment of future ecosystem problems, which will eventually enable for a timely intervention, before the impacts are visible and irreversible.

**Figure 1:**
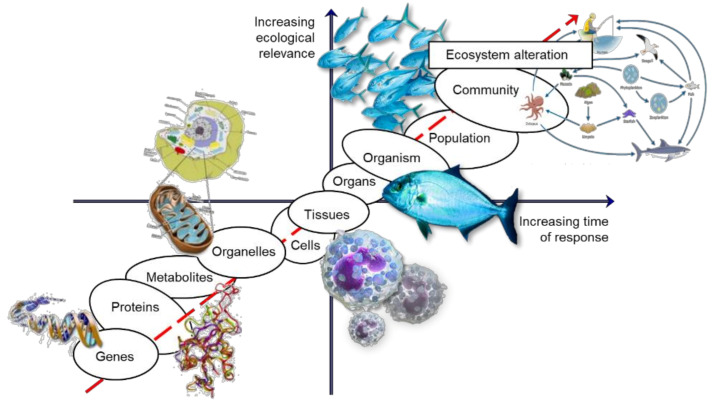
The biological organization continuum, relationship between temporal scale of response and ecological relevance after stress exposure (adapted from Lemos et al. 2010 [9]).

However, despite providing an early warning and an increased knowledge of the toxicity mechanisms, allowing the protection of biological integrity, the major setback is that these endpoints may fail to foresee later impacts on the environment, due to ecosystem resilience or weak link to the effects in the following level of biological organization, making these tools just too conservative for stakeholder interests [11]. Hence, an approach to targeting lower levels will always require addressing the potential effects at higher levels of biological organization by establishing a link of biological organization where the effects assessed at the lower end of the biological organization axis (if of sufficient duration and magnitude) are linked, with a high probability of causing effects, to the other end, including to populations and communities, and eventually causing ecosystem alterations later in time [9] (Figure 1).

Within this framework, biomarkers arise as a resourceful sub-individual tool in ecotoxicology. As with many other techniques applied in environmental sciences, arising from health science as a “characteristic that is objectively measured and evaluated as an indicator of normal biological processes, pathogenic processes, or pharmacologic responses to a therapeutic intervention” [12] or a “chemical, its metabolite, or the product of an interaction between a chemical and some target molecule or cell that is measured in the human body” [13], biomarkers are presently well established to address the biological effects of environmental contamination.

Similar to everyday use of body temperature, used as a proxy for fever, or even cholesterol that is acknowledged as a biomarker of cardiovascular risk, many are the endpoints that may be considered in other disciplines other than public health, including environmental sciences, where the use of biomarkers was initially promoted in the 1990s.

The environmental biomarkers can then be defined as “any biological response to an environmental chemical at the below individual level, measured inside an organism or in its products (urine, faeces, hairs, feathers, etc.), indicating a departure from the normal status, that cannot be detected from the intact organism” [14], or following Depledge [15] defined as a “biochemical, cellular, physiological or behavioural variation that can be measured in tissue or body fluid samples or at the level of whole organisms, to provide evidence of exposure and/or effects from one or more contaminants”. Both definitions, for modern purposes, should replace “chemical agents” by “stressor” to include other biotic and abiotic features that impact organisms. They are used to characterize stressors’ mode of action, to establish cause–effect relationships, to point to the presence of a certain group of contaminants, and for environmental health monitoring [16]. As already identified, in environmental studies and decision-making, the use of these endpoints is frequently critiqued for their limited ecological relevance as of unknown ecological significance of many sub-cellular responses [9]. This means that a sole significant response of a sub-individual endpoint may prove to be meaningless (or of limited scope, solely indicating exposure) and the cornerstone challenge is to find relationships between these biomarkers and ecologically relevant parameters, and thus obtain the full power of biomarkers as endpoints with high probability to be early warning signals of what may happen in the future to the population/community, while also enabling us to dig deep into a stressor impact mechanistic understanding.

One of the most comprehensive examples includes the biomarker acetylcholinesterase (AChE; EC 3.1.1.7). This may be included in a group of neurotoxicity biomarkers which may give a sign of neuromuscular disorder when addressing the acetylcholine receptor-ion channel complex (AChR) of skeletal muscles. Briefly, the nervous impulse passes when the molecule acetylcholine travels through the axons from the presynaptic membrane to the postsynaptic membrane. At the postsynaptic membrane it connects to a receptor opening an ion channel and the nervous impulse passes through. When the enzyme acetylcholinesterase catabolizes acetylcholine in choline and acetate, the receptor-acetylcholine complex is broken and the nervous impulse stops. Thus, AChE acts as a key of this nervous impulse and has a paramount role as a neuromuscular mediator. As a case study on the use of this biomarker, one may recall to the work of Venkateswara Rao et al. [17] addressing the effects of organophosphates in the brine shrimp, *Artemia salina*. In this study, Chloropyrifos, Profenofos, Monocrotophos, and Acephate were used as exposure media in the laboratory, and AChE activity was evaluated. For all tested organophosphates, there was a significant inhibition of AChE. Notwithstanding, despite this activity inhibition, one can argue about what does it mean and what it might represent in the biomarker world and shout a “so what?”—as stated before, the low relevance of this level is generally weakly linked to any meaningful real ecological impact. To establish a probabilistic relation with relevant scenarios, this study’s authors monitored the brine shrimp’s behaviour in an arena through video-tracking, where endpoints such as distance travelled, and speed were recorded after exposure to the organophosphates. Authors found a significant reduction in distance travelled and speed in those brine shrimp exposed to the compounds. By linking the AChE inhibition to impaired behaviour, one may now argue about the usefulness of this biomarker, as this link through these levels of biological organization may imply that enzyme inhibition may lead to behavioural impairment and when an organism moves in an increasingly less coordinated way, it will diminish the chances to find food or escape predators (e.g.,) and thus having impacts on survival, growth or reproduction and thus the implicated effects on population numbers and dynamics.

With this example, relevance to a low level of biological organization is given by linking probabilistically to higher levels of biological organization, while maintaining the backbone of the biomarkers’ advantage; that is, to give earlier responses while providing a mechanistic overview of the effects.

The aforementioned example is transversal to a myriad of environments and taxa, with many other authors also establishing such model links, such as with the bivalve *Corbicula fluminea*, exposed to the pyrethroid insecticide bifenthrin [18], or the common prawn *Palaemon serratus* exposed to deltamethrin [19], or the freshwater *Daphnia magna* exposed to chlorpyrifos [20], or the coho salmon *Oncorhynchus kisutch* exposed to chlorpyrifos [21], or even in the terrestrial environment the earthworm *Eisenia fetida* exposed to atrazine [22], or the carabid beetle *Pterostichus cupreus* [23], or the common shrew *Sorex araneus* [24], exposed to dimethoate, just to give few examples and all linking AChE to behaviour and establishing consequent causal effects at higher levels.

Still, much can be discussed and studied about the degree to which a given impact at a certain level will increase the chance of having effects also at the following level—and this is paradigmatic for biomarkers, as a whole, and definitely one of the biggest challenges for its use for regulatory purposes.

## 3. The Use of Biomarkers

When thoughtfully used, biomarkers may often serve as early-warning tools of adverse effects, and may also detect integrated effects from exposure to complex mixtures, integrate exposure events in time and space, help identify possible mechanisms of toxic action and calculate the exposure magnitude, more rarely identify the chemicals causing the effects [25], and ultimately, once the sample is processed and in a tube, with due adaptations, endpoints may be used in a vast array of taxa, organs, environments and situations.

The myriad of biomarkers—the number of which is only limited by the knowledge of the organism’s physiology and potential responses—may be divided into biomarkers of susceptibility, as an organism’s natural characteristic that make it more susceptible to the impact of a chemical, biomarkers of exposure, that represents the actual chemicals, or metabolites, being measured in the body or after excretion, and biomarkers of effect, which are quantifiable changes in an individual, indicating an exposure to a chemical which may translate into an effect on the organism’s health [26].

The latter may also be divided into protective and non-protective biomarkers, which by simple interpretation and definition distinguishes a non-protective as a biomarker that is assessed as a measure of the impact in the organism, such as a damage (e.g., lipids or DNA damage) or a protein inhibition (e.g., AChE), while a protective biomarker is one which demonstrates the organism’s reaction to diminish the impact of the stressor (e.g., antioxidant enzyme) [27].

Nowadays, and if one may speak of classic biomarkers, it is acknowledged that we may target functional endpoints included in major assemblages such as neurotransmission, oxidative stress, detoxification, energetic, immunological, or even reproductive, and concomitantly, every approach implies a limited group of functional responses and an a priori hypothesis about the predicted potential effects of a given stressor.

A simple search for manuscripts on the topic was performed in the SCOPUS database using the following word combinations: (ecotoxicology) AND (biomarker*) in the field “article title, abstract, keywords”. This resulted in a total of 2283 documents available until 27 October 2021. From these an extra “article title, abstract, keywords” field was added combining independently the words (neuro*), resulting in 203 documents; (detox*), resulting in 176 documents; (oxidative), resulting in 611 documents; (energ*), resulting in 155 documents; (immun*), resulting in 248 documents; and (reproducti*), resulting in 247 documents. Considering the apparently most addressed biomarker, the following section will further depict oxidative stress as a biomarker case study.

## 4. Oxidative Stress as a Cornerstone Example

Everyday course or merely living induces metabolic processes which produce reactive oxygen species (ROS), including reactive molecules and free radicals with molecular oxygen origin. These molecules are by-products of the aerobic respiration mitochondrial electron transport or are originated by oxidoreductase enzymes and metal catalysed oxidation and have been demonstrated to have an intra- and intercellular signaling role such as gene expression, the activation of cell signaling cascades, and apoptosis [28,29], and have traditionally been pointed as phagocytic cell responses to microbial invasion. Oxygen’s electron structure makes it prone to radical formation. The sequential reduction of oxygen through the addition of electrons leads to the formation of several ROS, including hydrogen peroxide, hydroxyl ion, hydroxyl radical, nitric oxide, and superoxide. These ROS, due to their chemical nature, have the potential to cause damage to several compartments in the organism, such as lipids, DNA or even proteins, and may ultimately lead to irreversible damage and even death.

**Figure 2:**
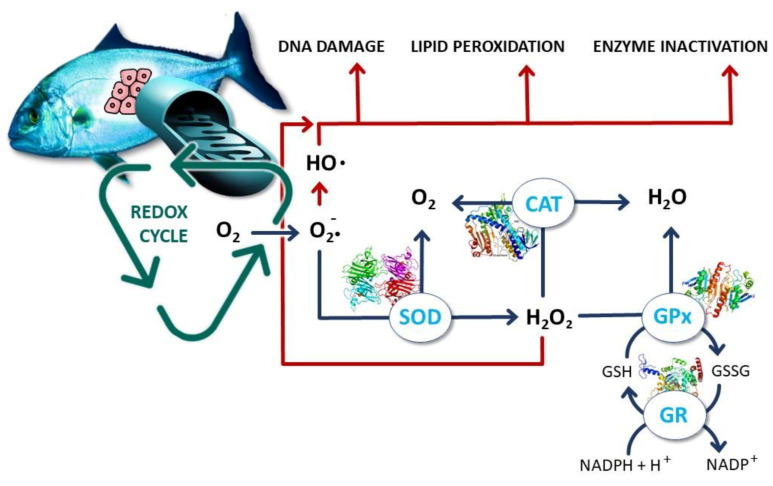
Schematics of mitochondrial reactive oxygen species formation, oxidative stress, detoxification, and cellular damage. CAT: catalase; SOD: superoxide dismutase; GPx: glutathione peroxidase; GR: glutathione reductase; GSH: reduced glutathione; GSSG: oxidised glutathione. Red lines represent a damage pathway and blue lines represent the detoxification pathways.

When ROS are produced in a higher amount than their removal, the state of oxidative stress happens. Despite the potential toxicity of ROS to living organisms, these may pose only relative issues as there are intrinsic cellular defences which enable an extent of detoxification of these molecules (Figure 2).

Superoxide dismutase (SOD; EC 1.15.1.1) catalyses the conversion of two superoxide anions into hydrogen peroxide molecule (H_2_O_2_) and oxygen (O_2_). In the peroxisomes, the enzyme catalase (CAT; E.C. 1.11.1.6) converts H_2_O_2_ into water and O_2_, completing the detoxification process started by SOD. Glutathione peroxidase (GPx; EC 1.11.1.9) is a group of enzymes containing selenium which catalyse the degradation of H_2_O_2_, and organic peroxides to alcohols [30]. To complement these, there are also non-enzymatic small molecule antioxidants that play a role in detoxification, like vitamin C (ascorbic acid) that is a water-soluble molecule able to reduce ROS, vitamin E (α-tocopherol), a lipid soluble molecule suggested to play a similar role in membranes, and glutathione [31]. This latter, a tripeptide (glutamyl-cysteinyl-glycine), may be the most important intra-cellular defence, with an exposed sulphhydryl group, which may serve as a target for ROS attack, oxidizing glutathione (GSSG), while the reduced form (GSH) is then regenerated by a NADPH-dependent reductase [31]. This GSH/GSSG ratio is thus used as a dynamic indicator of an organism oxidative stress [32].

As mentioned, the created ROS/defences imbalance establishes an oxidative stress environment, capable of attacking vital molecules with impacts that will depend on the magnitude of the unbalance. Unsaturated fatty acids like those present in cellular membranes are common targets, being lipid peroxidation (LPO) one of the most used biomarkers for free radical formation, because of chain reactions in which a free radical captures a hydrogen from an unsaturated carbon to form water, leaving an unpaired electron capable of capturing oxygen and forming a peroxyl radical [28]. These lipid peroxides due to their instability decompose and form a complex series of compounds, including the reactive carbonyl malondialdehyde (MDA), which is the target of most methodologies as a proxy of LPO [28]. Additionally, this ROS unbalance represents a hazard to DNA as they modify bases (especially guanine, due to its oxidation potential) and might disrupt genome function, induce genome instability and mutation [31]. If exposure rate outperforms intrinsic repair process, it will result in processes such as inflammation, ageing, and the development of multiple age-related diseases, such as neurodegenerative disorders and cancer [28]. This damage is most often assessed by addressing DNA strand breaks, either by using a comet assay technique [33] or a more high-throughput colorimetric assay [34]. The whole of oxidative damage may not represent solely a structural damage to the organism, but may in fact, alter metabolic functions by affecting proteins, which of course includes enzymes that, as mentioned, are involved in the detoxification process of ROS. In this case, a higher production of ROS and incapacity to cope with these high levels may ultimately inhibit enzyme activity and worsen the capacity of enzymes like CAT, SOD, or GPx to neutralize the ROS, which often leads to non-monotonic responses—with an increase of these enzymes to cope with stress and then a decrease due to protein damage at higher concentrations [35]. The non-monotonicity of responses adds extra complexity to the interpretation of results and concentrations addressed, this being a comprehensive example of the challenge to interpret isolated biomarker responses [36].

## 5. Two Is Better Than One, but the More the Merrier

This latter issue represents one of the biggest challenges an ecotoxicologist will have to face when dealing with the interpretation of the selected biomarker responses. Unspecific stress/response biomarker (excluding few, such as lead specific delta-aminolevulinic acid dehydratase (ALAD), for example), non-monotonic dose-responses (often translated in hormesis or low-dose effects) and choosing a limited array of biomarkers for a hypothesis-driven approach, will give an incomplete and many times misleading view of toxicological mechanisms. To better understand something that is in fact a complex puzzle, one must choose a set of pieces that will give a clearer view of what may be happening in response to a given stress, setting up a story with a less biased mechanistic view. For instance, concerning oxidative stress, if one decides to choose one enzyme as a proxy, e.g., CAT, one will be looking for an increase in case of oxidative stress. Nevertheless, this might not be the case as the organism may be using other enzymes rather than CAT for the process (several organisms may not even possess this enzyme or few, e.g., Rato et al. [37]) or an apparent normal or decreased activity might be the consequence of protein damage or production issues derived from severe systemic toxicity [34]. This is the framework where the need for a decision to choose from a limited, yet diverse array of biomarkers is paramount. For the stated example, beside choosing CAT, addressing SOD, GPx (and/or others) and damage such as LPO or DNA damage will offer a more comprehensive view, and as more biomarkers are added the clearer the puzzle will be, which may include other endpoints related to oxidative stress. Additionally, besides other considerations due the stress specificity where one can include a specific target set of biomarkers, since the increase in the mechanisms to tackle and survive stress do not come without a cost (“cost of tolerance” theory) [38], to analyse energetic biomarkers may be deemed important to depicting energetic trade-offs and changes in the normal repartition of organisms’ energy [39,40,41], allocating it to stress response mechanisms in order to cope with the induced stress. Cellular energy allocation (CEA) is a methodology used to assess and quantify stress-induced energetic trade-offs in the organism by integrating the energetic reserves proteins, lipids, and carbohydrates, as components of the energy available, divided by the energy consumption in the cellular metabolism at a given time, determined based on the measurement of the maximum potential activity of the mitochondrial electron transport system (ETS) in the respiratory chain [8]. An increase of stress should increase ETS and concomitantly reduce the energy available and thus energy potentially used for somatic and germinative growth. Of great usefulness in the energetic realm is also the measurement of the ratio of metabolic key aerobic metabolism enzyme lactate dehydrogenase (LDH; EC 1.1.1.27) and anaerobic metabolism isocitrate dehydrogenase (IDH; EC 1.1.1.41 and EC 1.1.1.42). Under stressful condition, organisms to mobilize more and faster energy for metabolic purposes will rely to a greater extent on the anaerobic path and thus this LDH/IDH will tend to increase which may then be used as a proxy for the metabolic use of energy [42]. Increased respiration will lead to cellular oxidative stress brought by accumulating ROS, adding significance to measuring a bundle of these stress-related biomarkers to enlighten stress effects.

By integrating different classes of biomarkers, altogether they will offer a more integrative analysis and with an expected increase of relevance. Nonetheless, this decision on the biomarker targets will be the result from a hypothesis-driven approach, bearing in mind what may happen and what the researcher aims at with the study. On the other hand, a non-hypothesis driven approach, where the overall view and expected and non-expected mechanisms are targeted, may suit a distantiation and better search for the unknown and intrinsic complexity of interconnected responses, but most often decreasing method sensitivity and increasing the challenge and risk of falling into too much indigestible information [9]. A more complete array of explanatory non-hypothesis driven tools such as a vaster selection of target biomarkers with higher throughput techniques like OMICS [35,43,44,45] or the fatty acid profile [34,46,47,48,49], e.g., may help us to hold a comprehensive knowledge of multipart biological processes as integrated systems rather than an assembly of isolated parts, but comes with an array of associated challenges and complex methods falling beyond the scope of this work.

## 6. Understanding Biomarker Communication

As mentioned, selecting a wider array of biomarkers provides a more complete toxicological mechanistic assessment, and statistical tools have been created to sum up the overall biomarker impact of a given stress on the organism, enabling us to compare the total magnitude of response, irrespective of the mechanism, as well as to simplify information making its communication easier, especially to the non-scientific community [50]. A collection of scoring indexes has thus emerged [51] that, following biomarker evaluations and other suitable endpoints, transforms the collections of biochemical data into numeric indexes to accurately reflect the integrity and emphasize the biological “health status”, and simplify result communication [51,52]. Such approaches are grounded on different uses of the same biochemical data, including indexes such as the bioeffect assessment index [53], the multi-biomarker pollution index [54], the biomarker response index [55], the principal component analysis scoring-based index (PCA-index) [56], or the integrated biomarker response index (IBR) [57]; this latter used presently as an example as it is one of the most proficient and widely used.

**Figure 3:**
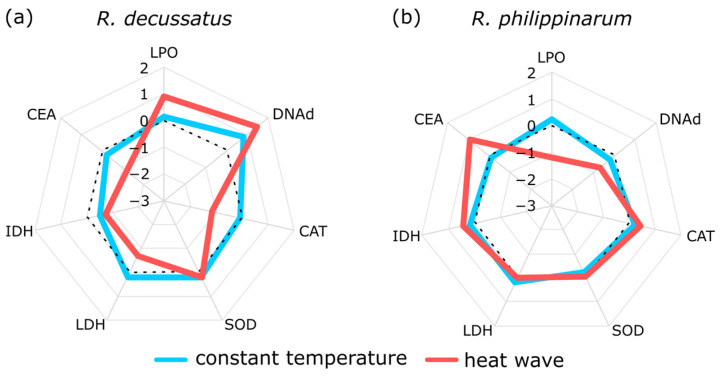
Biomarker integrative star plots measured in (**a**) *Ruditapes decussatus* and (**b**) *R. philippinarum* at constant temperature and after a heat wave simulation: SOD–superoxide dismutase activity; CAT–catalase activity; LPO–lipid peroxidation levels; DNAd–DNA damage; CEA–cellular energy allocation; LDH–lactate dehydrogenase activity; and IDH–isocitrate dehydrogenase activity (adapted from [58]).

In Crespo et al. [58], to understand how global changes anomalies (increase in temperature) may contribute to the success of biological invasions, two closely related species, the native *Ruditapes decussatus* and the invasive *R. philippinarum*, were exposed to a simulated similar heat wave. Besides behaviour/bioturbation, as an ecological process, biomarkers such as SOD, CAT, LPO, DNA damage, IDH, LDH, and CEA were assessed. The results pinpointed a better energetic condition of *R. philippinarum* to cope with thermal stress, namely through the increase in lipid reserves and cellular energy allocation, and decrease of LPO, which may improve its fitness and success in an invasion scenario (for depicted results see Figures 3–5 in Crespo et al. [58]). Nevertheless, despite the mechanistic understanding, the use of several biomarkers will not give an overall quantifiable answer about which species will perform better under these climatic events. In this context, the use of the IBR index allows to integrate these biomarkers, after data normalization to overcome the differences in the magnitude between the different biomarker traits and achieve an individual biomarker score and a star plot representation (Figure 3), granting improved support by showing an increase of damage and energy reserves in the native and an increase of reserves and less damage in the invasive.

**Figure 4:**
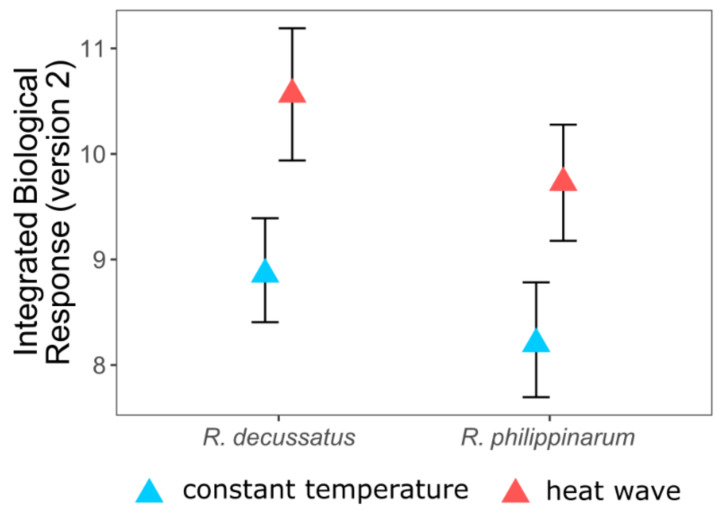
Integrated biological response of *Ruditapes decussatus* and *R. philippinarum* exposed to different temperature treatments (adapted from [58]).

The analysis of the biochemical biomarkers, through the IBR index (following Sanchez et al. [59]), allowed an integrated assessment of the susceptibility of bivalve species to global changes, summing it up in a global score (Figure 4), where the impact of the heat wave is made clear for both species, by an overall increase of the IBR value, but lower for the invasive.

The application of IBR appears as a useful simplification tool to identify stress situations, reflecting the overall set of data, despite the potential variability of biomarkers used for its calculation, presenting a comprehensive and robust scoring framework, where low values represent a lack or less stress and high values represent higher environmental stress, which in all cases is a rather clear way to present results to a less specialized community [50].

## 7. Applications and Perspectives

The ultimate goal for the use of biomarkers is to have a predictive tool that may give a mechanistic overview of the impact in a given organism but, more importantly, that may provide insight about the potential impacts in the organism’s fitness and cascading events up to the populations and communities, in order to protect ecosystems’ sustainability. There are many challenges concerning their application and that have been addressed, slowing down the ability to leverage these from the lab bench to applications in real scenarios.

Despite being recognized as potential tools for environmental risk assessment [55], they have not yet been incorporated in regulatory legislation, in particular in the Water Framework Directive (WFD) recommended procedures, or in the Biocides or Plant Protection Products Directives. Factors like temperature, reproductive cycles, seasonality, gender, age, life-stage, among many others may affect biomarker responses (e.g., [25]) and hamper their interpretation for many purposes. Nonetheless, when one understands the nature of such limitations and that not all biomarkers are useful for a particular need, and when a well-planned experimental design is used, biomarkers may be used as reliable and sensible tools with a great explanatory potential. Its usefulness is thus unquestionable.

By understanding the individual effects and linking them to the higher levels, laboratory experimentation may allow us to understand current issues and build future probable scenarios. For example, by exposing the two-spotted goby, *Gobiusculus flavescens*, to high pCO_2_, Faria et al. [60] was able to link a trade-off of energy allocation with fish reproductive success, while Silva et al. [61] linked oxidative stress and differential energy allocation to impacts in larval development in sand smelt larvae, *Atherina presbyter*, exposed to high pCO_2_. Also, Silva et al. [47] found that the damage induced to the sand smelt larvae’s DNA after exposure to high pCO_2_ water was not reversible even after being transferred to control conditions, which may threaten species survival. Also related to ocean acidification and climate change, Rato et al. [37] tested the effects of acidification on *Homarus gammarus* larvae and found they suffered from oxidative stress while they also presented developmental impairment, namely decreasing their growth. All these examples, to name a few, point towards the impacts that Intergovernmental Panel on Climate Change (IPCC) estimates for ocean acidification by 2100, and to the usefulness of biomarkers in providing an insight into future global change issues, with worrying information about species’ capability to cope with these changes. Besides acidification, temperature changes are a key issue for climate change, which has also been studied addressing biomarkers. In research on the rock pool fish *Gobius paganellus*, Paul et al. [42] found that the increase of temperature led to decreased growth, increased LDH/IDH ratio (due to increased anaerobic metabolism), decreased ETS, and increased DNA damage. They also found that predation risk impacted this organism’s cellular metabolism, but the severity of effects of thermal stress clearly outweighed metabolism-related responses to predation stress increasing the risk of being preyed upon and inducing an extra energy trade-off for the basal metabolism, which may have ecologically relevant consequences. These two latter studies are examples of how the study of biomarkers may give insights into predicted future global temperature changes.

Likewise, by allowing an understanding of how organisms respond to different environmental situations and their trade-offs that may relate to the capability of a population to thrive under those conditions [49,62,63], biomarkers may further provide important information about how species will cope in an introduced environment and how they will compete with native species for a given niche, under a current or changing environment, and potential adaptation and spread, disclosing potential invasive setups [58,64,65]. Also, and regarding biological invasions, studies have been reported on the impacts of the invasive species on native species biomarker endpoints which may lead to a community impairment and invasion success, as in the case of *Asparagopsis armata*, a red seaweed producing halogenated compounds and releasing them into the environment and with ecosystem impacts [34], namely survival, and also reported impacts on macrofauna energetic biomarkers [40], and oxidative stress and damage, neurotoxic effects, and inflammation and immune responses [34], limiting the number of foraging herbivores and creating a thriving environment for this species and modulating the landscape and biodiversity.

Evidently, most of the uses of this biomarker tool goes to laboratorial assessment of biotic and abiotic factors, but a big share of studies account for the impacts of global changes on wildlife, with applicability in fishes (e.g., [66,67,68]), birds (e.g., [69,70]), turtles (e.g., [71,72]), or terrestrial and marine mammals (e.g., [73,74]), to name a few. The use of biomarkers is not limited to the realm of environmental sciences, that is a fact, and is (and should be) used increasingly to understand mechanisms that can lead to a better commercial use of organisms. Aquaculture is one niche where the use of biomarkers has been used to address stress upon reared species, owing to the known fact that trade-offs may be disastrous in a production site—energy allocated to face stress will not be used for growth and reproduction—and to increased awareness of animal wellbeing. Considering this latter issue, studies may address stress biomarkers (such as oxidative stress markers of energetic endpoints) to understand best practices for aquatic organism handling, transportation, and acclimation and quarantine (e.g., [75]), which normally are addressed by verifying injury or death, which is in fact a later stage of distress and does not account for animal welfare. To maintain the best somatic and germinative growth, the best conditions should be maintained, and this includes balanced and optimized feeds that may not only provide best nutritional value but also best performances, which can be addressed through fatty acids or oxidative stress biomarkers (e.g., [46,76,77]). Even when focusing on new biotechnological challenges for aquaculture feed and reducing mortality when facing severe pathologies, biomarkers can be valuable tools to evaluate fitness, as in the example of the study by Félix et al. [78] where shrimp resilience against a known *Vibrio* pathogen was assessed after the inclusion of different seaweed extracts in their feed, concluding about the efficiency of these novel feeds by addressing histopathological biomarkers on the shrimp’s hepatopancreas in order to evaluate possible damage caused by *V. parahaemolyticus* infection.

The possibilities for the use of biomarkers in the stress biology realm are virtually countless and will continue to overcome boundaries as long as an organism’s physiology and ecology are well known and while high throughput tools are continuously developed along with strong statistical and informatic tools, and yet, most importantly, gaining more importance the better we establish the link between the sub-individual level response and high levels of biological organization. This will, after all, give relevance to the tool, proving its usefulness in a myriad of applications.

## Data Availability

Not applicable.
